# Online Magnetic Resonance-Guided Radiotherapy (oMRgRT) for Gynecological Cancers

**DOI:** 10.3389/fonc.2021.628131

**Published:** 2021-08-27

**Authors:** Lorraine Portelance, Stefanie Corradini, Beth Erickson, Susan Lalondrelle, Kyle Padgett, Femke van der Leij, Astrid van Lier, Ina Jürgenliemk-Schulz

**Affiliations:** ^1^Sylvester Comprehensive Cancer Center, Radiation Oncology Department, University of Miami, Miami, FL, United States; ^2^Department of Radiation Oncology, University Hospital, LMU Munich, Munich, Germany; ^3^Department of Radiation Oncology, Medical College of Wisconsin, Milwaukee, WI, United States; ^4^Department of Clinical Oncology, The Royal Marsden NHS Foundation Trust and Institute of Cancer Research London, London, United Kingdom; ^5^Department of Radiation Oncology, University Medical Center Utrecht, University Utrecht, Utrecht, Netherlands

**Keywords:** gynecological cancers, MR-guided radiotherapy, MR Linac, SBRT, cervical cancer, online MR guided radiation therapy

## Abstract

Radiation therapy (RT) is increasingly being used in gynecological cancer management. RT delivered with curative or palliative intent can be administered alone or combined with chemotherapy or surgery. Advanced treatment planning and delivery techniques such as intensity-modulated radiation therapy, including volumetric modulated arc therapy, and image-guided adaptive brachytherapy allow for highly conformal radiation dose delivery leading to improved tumor control rates and less treatment toxicity. Quality on-board imaging that provides accurate visualization of target and surrounding organs at risk is a critical feature of these advanced techniques. As soft tissue contrast resolution is superior with magnetic resonance imaging (MRI) compared to other imaging modalities, MRI has been used increasingly to delineate tumor from adjacent soft tissues and organs at risk from initial diagnosis to tumor response evaluation. Gynecological cancers often have poor contrast resolution compared to the surrounding tissues on computed tomography scan, and consequently the benefit of MRI is high. One example is in management of locally advanced cervix cancer where adaptive MRI guidance has been broadly implemented for adaptive brachytherapy. The role of MRI for external beam RT is also steadily increasing. MRI information is being used for treatment planning, predicting, and monitoring position shifts and accounting for tissue deformation and target regression during treatment. The recent clinical introduction of online MRI-guided radiation therapy (oMRgRT) could be the next step in high-precision RT. This technology provides a tool to take full advantage of MRI not only at the time of initial treatment planning but as well as for daily position verification and online plan adaptation. Cervical, endometrial, vaginal, and oligometastatic ovarian cancers are being treated on MRI linear accelerator systems throughout the world. This review summarizes the current state, early experience, ongoing trials, and future directions of oMRgRT in the management of gynecological cancers.

## Introduction

As early as 1990, magnetic resonance imaging (MRI) was described as a promising tool in management of gynecological cancers providing superior visualization of tumor and adjacent pelvic anatomy compared to other imaging modalities (1). In 1992 Russell published a review that highlighted the potential for MRI guidance to avoid marginal tumor misses in external beam radiation therapy (EBRT) of gynecologic cancer (2).

A decade later, the use of MRI was introduced in the brachytherapy (BT) planning process for patients with locally advanced cervical cancer (LACC) (3). MRI-guided (MRg) BT is based on an adaptive target concept that accounts for the topography of the primary tumor at diagnosis as well as the regression observed during EBRT (4). There is now a large collection of literature demonstrating that image-guided adaptive BT (IGABT) leads to better tumor control, increased survival, and decreased treatment toxicity (5–9). IGABT is supported by both the Groupe Européen de Curiethérapie European Society for Radiation Oncology as well as the American Brachytherapy Society and several guidelines have been published (3, 4, 10).

MRI is steadily gaining importance for diagnostic purposes and for optimizing the radiation treatment of gynecological malignancies (11). It has become a key component of initial disease staging for cervix cancer (12), and MRI findings are now integrated in the International Federation of Gynecology and Obstetrics (FIGO) cervical cancer staging system. MRI has been adopted as the imaging modality of choice for the management of patients with cervical cancer due to superior soft tissue contrast compared to computed tomography (CT). This allows for better visualization of the pelvic and abdominal organs and better distinguishing tumor from adjacent healthy tissues. Sequential MRIs during EBRT can capture inter- and intra-fraction motion, deformation of the tumor and the surrounding organs, and tumor regression over time (13, 14).

The integration of an MRI in a linear accelerator (MR Linac) treatment unit (Unity, Elekta, Sweden; MRIdian, ViewRay, Cleveland, OH, USA) constitutes a real breakthrough for the management of gynecological malignancies, allowing physicians to perform online adaptive radiation therapy (ART) based on the anatomy of the day and to monitor anatomical changes during a treatment course. Utilizing ART, new strategies are being developed to increase EBRT conformality and further individualize treatment plans. Treating gynecological malignancies with an online MRg radiation therapy (oMRgRT) approach has the potential to reduce treatment toxicity and optimize tumor control, which would be consistent with IGABT results.

Patient selection depends on patient characteristics and disease characteristics. Patients could be physically incompatible for oMRgRT based on the presence of non-MRI compatible cardiac implantable electronic device, or any other type of metallic implant/foreign bodies or clinically incompatible, for example, patients suffering from claustrophobia, severe anxiety, pain preventing them from being able to hold the same position for a long time on the treatment table (the whole replanning, treatment delivery process might be up to 60 min). In terms of disease characteristic, there is a large spectrum of gynecological cancers that might benefit from oMRgRT. In the curative treatment of cervical cancer, oMRgRT may be utilized for elective EBRT nodal boosts and primary tumor boosts if first-line BT is not feasible. Patients with gynecologic cancers who might also benefit from oMRgRT include those with locoregional recurrences after surgery and those with oligometastatic who are no longer responding to systemic therapy or are not candidates for systemic therapy due to the presence of comorbidities (15). For the latter group, oMRg stereotactic body radiation therapy (SBRT) could be applied to both nodal and soft tissue metastasis to achieve target tumor control with limited morbidity. SBRT of oligometastatic disease has been reported to increase survival while preserving quality of life (16).

In this manuscript we review early clinical applications of oMRgRT and its use for various gynecologic tumor sites and with different treatment intents and reflect on current hypotheses supporting the use of oMRgRT in gynecologic cancers.

## Treatment of Locally Advanced Cervical Cancer

Definitive treatment of LACC consists of EBRT to the primary tumor, the entire cervix and uterus, the parametria, the upper vagina, and draining lymphatic regions along with nodal boosts to positive nodes usually combined with chemotherapy (mostly weekly cisplatin). Elective paraaortic (PAO) nodal irradiation may be indicated in some patients. It is standard of care to deliver a BT boost to the residual primary tumor after EBRT. BT and EBRT both benefit from MRI guidance, but in different ways.

With modern radiation therapy (RT), daily verification for target positioning has improved significantly. Since the 1990s, EBRT has evolved from the use of Port films and skin marks to the use of cone beam CT (CBCT) with or without fiducial markers for more precise targeting of soft tissue lesions. Daily on-board image guidance has become standard of care, but the suboptimal soft tissue contrast provided by CBCT makes it challenging to distinguish soft tissue tumor from surrounding normal tissues, particularly in the pelvis.

MRI provides superior soft tissue contrast compared to CT. As opposed to CBCT, there is no additional ionizing radiation exposure when MRI is used for on-board daily imaging. Ultrasound imaging can also provide a low-cost, non-ionizing radiation verification tool in LACC radiotherapy (18) and can be linked with treatment delivery. However, whilst the uterus, cervix, and bladder can be identified reliably, other OARs are not easily visualized.

MRI is already integrated into the radiation treatment planning pathway for LACC. In addition to providing better soft tissue resolution, MRI has the advantage of allowing depiction of disease extent in more than one plane (17). The possibility to perform image acquisition in two orthogonal planes along the tumor axis provides important information on disease extent for cervical cancer staging.

The BT literature has demonstrated the pivotal role of MRI in improving delineation of the high-risk clinical target volume (HR-CTV) (7) leading to better tumor control and reduced treatment toxicity (7–9).

### Adaptive Radiation Therapy in the Management of LACC

In the management of patients diagnosed with LACC, it is well known that the primary tumor exhibits large inter fraction motion due to day-to-day changes in the volume of the surrounding pelvic organs (mainly bladder, rectum, and other parts of the bowel) seen during the delivery of pelvic EBRT. Haripotepornkul et al. (18) calculated the inter-fractional movement of the cervix during intensity-modulated radiation therapy (IMRT) in the lateral, vertical, and anterior-posterior directions as 1.9, 4.1, and 4.2 mm, respectively. The simplest strategy used to deal with target inter-fraction and intra-fraction motion has been to add a generous planning target volume (PTV) margin of 1.5–2.0 cm to the target volume. This expanded security margin is necessary to ensure full dose to the target, but the cost of this approach is that a large part of the surrounding normal organs receives the same dose of radiation than the target volume.

Treating cervical cancer with ART can enhance precision during EBRT by correcting for the inter-fraction motion, thereby reducing PTV margins and the volume of non-target tissues that receive high-dose RT. Early exploratory studies on the use of oMRgRT demonstrated that daily MRI permits adaptation of EBRT plans to daily tumor and organs at risk (OAR) positions (14). The use of ART potentially leads to a considerable reduction in OAR dose, by facilitating improved accuracy of treatment delivery and enabling margin reduction.

A more recent comparative study of various ART techniques using CBCT with standard margins, reduced margins, and oMRgRT demonstrated that incremental dosimetric gains can be made in OAR sparing through the use of more advanced technology (19).

Another ART concept, only achievable with MR Linacs, challenges the convention of including the whole uterus in HR-CTV target volume. Contemporary consensus contouring guidelines for IMRT for cervical cancer advise including the whole uterus (20). These guidelines were written based on the limited ability of CT to identify intrauterine tumor extension. The safest way to deal with this uncertainty was to include the whole uterus in the initial target volume and to add a large margin on this volume to account for inter-fraction fundus motion. The ability of MRI to distinguish tumor from normal uterus introduces the possibility of targeting the tumor only rather than the tumor, cervix, and the whole uterus. A preliminary modeling suggests this is a feasible approach that could further reduce OAR dose (19). Kozak published a single institution retrospective study of 53 patients with LACC treated per institution policy with less than whole uterus irradiation volume and showed comparable locoregional control and reduced bowel V40 and D200cc when the outcomes from the cohort studies were compared to historical series (21). These preliminary data should not lead to broad clinical implementation but rather be seen as provocative results that deserve being tested in a larger multicenter international prospective study to confirm the safety of this approach.

Daily adaptive planning can significantly reduce treatment margins sparing surrounding OAR without compromising target coverage; however, these techniques are complicated, time-consuming, and resource intensive. Based on CTV-PTV margins of 3–5 mm, an online adaptive planning strategy can reduce dose to rectal V4000cGy by 36–47%, dose to bladder V4000cGy by 43–59%, and dose to bowel V4000cGy by 13–30% compared to a non-adaptive approach (19). As oMRgRT and auto-segmentation technology continue to improve, the burden of daily adaptive planning may be significantly reduced. Until these gains are realized, daily adaptive planning for cervix cancer may be impractical. However, a practical approach to mitigate the large treatment margins necessary for accounting for inter-fraction motion is to utilize a plan-of-the-day (POTD) technique (22).

**Figure 1:**
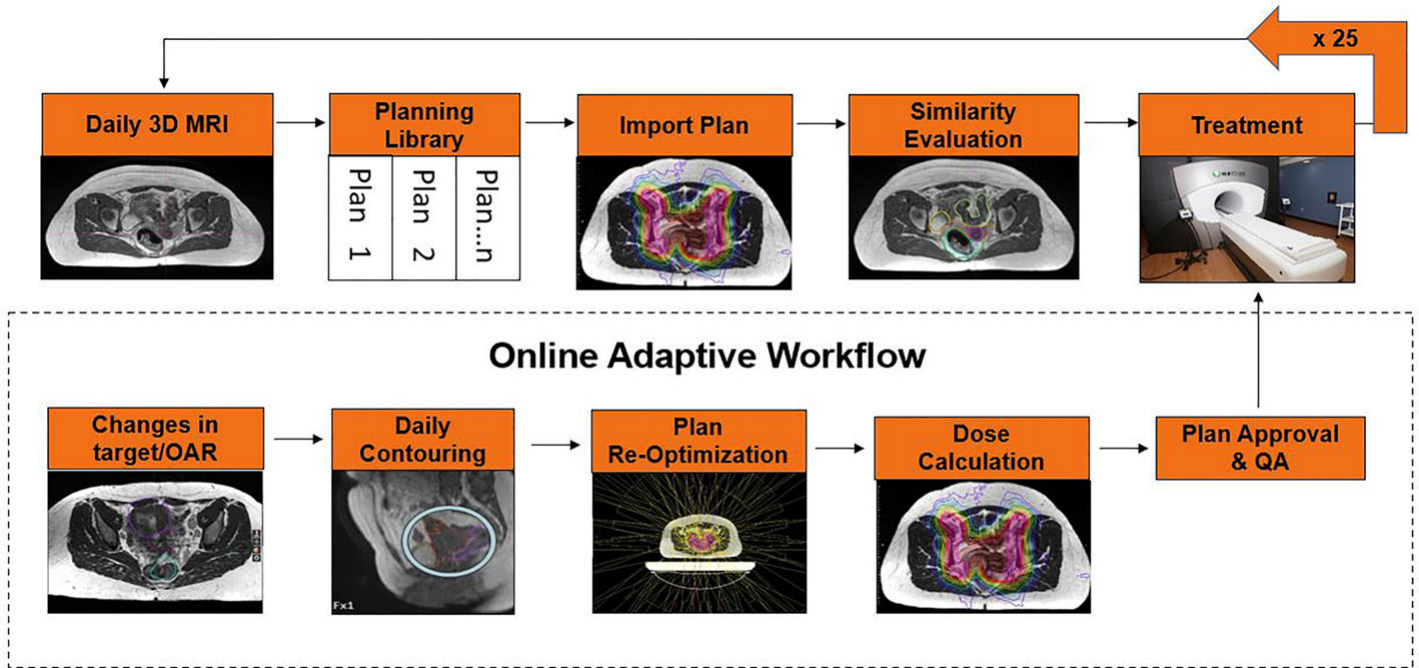
POTD approach for oMRgRT for cervical cancer.

The POTD technique utilizes an individualized IMRT plan library that is selected based on the patient’s internal anatomy at the time of daily setup. POTD technique has the potential to reduce the treatment margins compared to conventional treatments, but it has a more manageable workload and faster treatment time compared to daily ART. Buschmann et al. published their experience with 16 patients using a volumetric modulated arc therapy (VMAT) plan library for bladder full, bladder empty, and a motion robust backup plan, where plan selection is based upon daily setup CBCT. MR Linac systems could use a similar methodology but have the added benefit of creating the plan library as needed on fractions that do not have a suitable match in the current library for the patient, resulting in an adapted plan for the day and an additional entry into the plan library (**Figure 1**). Additionally, the improved MRI image quality compared to CBCT image will ease plan selection for those fractions where a predefined plan will suffice.

Although using oMRgRT for LACC has been seen as one of the key examples for using the ART approach with MR Linacs (given the good MRI visualization and large inter-fraction motion), to date clinical implementation of this treatment is limited. The main drawback of the currently available MR Linacs systems is the limited treatment field size [feet/head extent: 22 cm (Unity/Elekta), 24.1 cm (MRIdian/ViewRay Cleveland OH)], which especially hampers treatments that include elective treatment or nodal boosts, which could extend up to PAO nodes. Technically, a multiple isocenter approach may solve this; however, long treatment times, added treatment planning complexity (which might be challenging to safely integrate in an online planning workflow), and the risk of irradiating the same volume twice (especially the bowel) are to be considered in implementing this technique. Solutions including VMAT and tomotherapy approaches might provide additional gain.

Hypofractionation approaches could also be a practical solution to make oMRgRT workable. As it has been shown for other pelvic tumors (23, 24), the need for strict adherence to prescriptions of 1.8–2 Gy per fraction when treating the central pelvis plus nodes can be challenged. Hypofractionation used to be considered a safe approach only for small-volume targets, but there is growing acceptance that larger volumes can be treated similarly, provided doses to the more sensitive OAR such as bowel can be minimized. Studies exploiting the benefits of integrated MR Linacs for enhanced target and OAR visualization and online adaptation to treat LACC with hypofractionated schedules are in progress (25) and if successful will facilitate the wider adoption of daily replanning for cervical cancer.

### Potential Gain of oMRgRT When Brachytherapy Is Not Feasible

oMRgRT can also be used to substitute the final BT boost in selected cases (e.g., patients with comorbidities limiting their capacity to undergo invasive procedures, BT implantation technically not feasible). The first experience with this novel treatment approach has been published (26, 27). Due to the limited dimensions, delineated volumes, and number of fractions, this treatment option is easier to implement than treatment of longer EBRT fields. Compared to BT, however, with oMRgRT the target dose will be limited if isotoxic OAR constraints are used (27, 28). Focus on the OAR constraints is important, which is exemplified by the high toxicity reported in one study (29) in which relatively high OAR doses were allowed. Strictly using the current recommended BT OAR dose constraints for MR Linac SBRT treatments may be a good starting point to prevent high toxicity. In such an approach the OAR dose is driving the choices in treatment planning, and it can be expected that daily online re-planning with MR Linacs may deliver less dose to the targets compared to BT, but more target dose can be expected compared to CBCT-guided treatments (26). It was demonstrated in the BT literature that adhering to high-dose levels to the HR-CTV is critical to obtain local control (LC) (30, 31). As current studies show that the target dose is reduced using MR Linac treatments compared to BT (26, 29), and the efficiency of SBRT is still considered limited (32), BT remains the superior treatment option. A SEER review published by Eiffel has clearly demonstrated that the use of BT in the management of patients with LACC is associated with improved survival (33). MR Linac treatments should not be considered a replacement for BT, but it could be an option in selected cases where BT is not possible and, in these cases, might be preferable over CBCT-guided SBRT. A typical example is provided in case 1 below.

**Figure 2:**
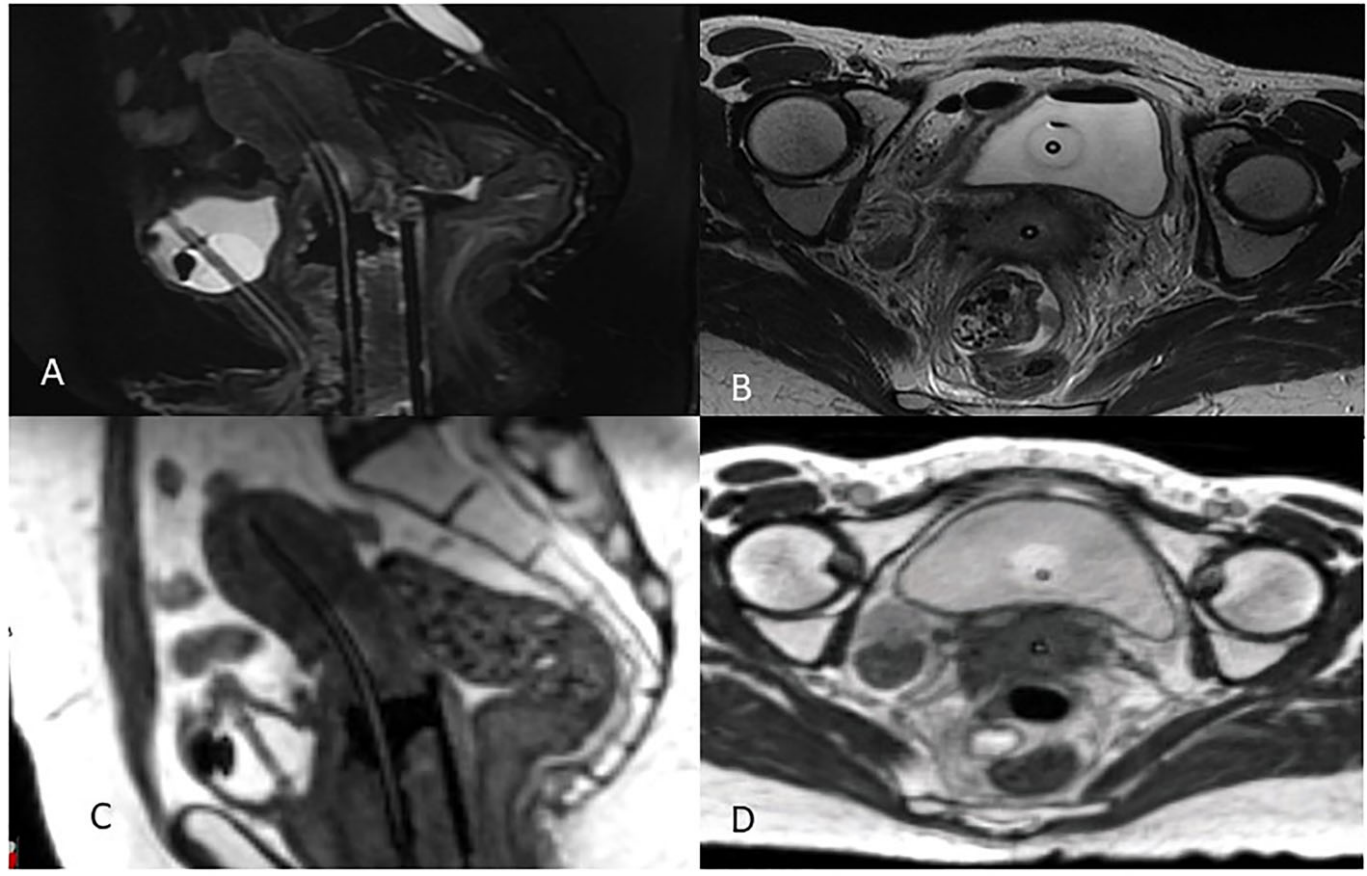
MRg Brachytherapy 3T Diagnostic MRI **(A)** Sagittal and **(B)** axial views compared to 0.35T MRIdian MRI **(C)** Sagittal **(D)** axial views.

The availability of an MR Linac treatment unit in the Radiation Oncology clinic has the additional benefit of providing easy access to MRI datasets with applicators in place to aid in MRg BT planning. This can greatly simplify the logistics of doing IGABT for many institutions who until now had relied on the limited availability of MRI scanners in the diagnostic radiology department (34). **Figure 2** is an example of a BT MRI study obtained using a 0.35 Tesla system. After immobilizing the applicators with a clamp or other MR-compatible device, the patient is transferred to the MR Linac room on an MR-compatible stretcher.

### Functional Imaging and Dose Painting

An additional appeal of integrated MR Linacs is the ability to perform serial functional imaging through the course of EBRT. The information obtained might be used to guide decisions on boosting poorly responding targets or as a prognostic tool to define the need for additional therapies. Diffusion weighted imaging (DWI), where random Brownian motion of water within tissues is detected, is currently used to determine malignant from benign tumors by measuring apparent diffusion coefficient (ADC) values. Malignant tumors exhibit a low ADC value and in combination with T2WI are highly sensitive in delineating tumor from surrounding tissues. Studies have demonstrated that serial ADC measurements during the treatment course can be used as an independent prognostic factor for treatment response, where increase in ADC values during treatment represents tumor response, thus aiding in identification of good responders (35, 36). DWI also demonstrates heterogeneity within the tumor, indicating areas of resistant clones as well as regression. With automated contouring, thresholds can be set for ADC values, and these areas could be targeted with a “dose painting” strategy—a concept whereby different doses can be delivered within the tumor.

Feasibility of using diagnostic DWI on the MR Linacs has been demonstrated, but reproducibility across systems and institutions is challenging due to inconsistent hardware and acquisition methods. With MR Linac institutions working in collaboration, work can be undertaken to identify appropriate sequences that can be applied across all machines, which will allow for reliability as well as repeatability. This collaborative approach, fostered in cervical cancer through the EMBRACE network, has been replicated in a sub study, iEMBRACE, which is currently investigating the use of serial functional imaging on diagnostic platforms as a prognostic tool in cervical cancer. The first step to standardize measurements across institutions has been successfully implemented.

Current research, using sequences acquired on MR Linac, will investigate the potential of other functional MRI sequences to measure tumor and normal tissue response (e.g., dynamic contrast enhanced (DCE) MRI).

## Treatment of Inoperable Endometrial and Recurrent Gynecological Cancer

### Inoperable Endometrial Cancer

The standard treatment of localized endometrial cancer is surgery consisting of hysterectomy with bilateral salpingo-oophorectomy with or without regional lymph node dissection or sentinel lymph node mapping. This treatment may need to be followed by radiotherapy and/or systemic treatment depending on histopathologic risk factors. A minority of patients are unable to undergo surgery due to advanced age, poor performance status, or medical contraindications to anesthesia. These patients can be treated with definitive radiotherapy consisting of EBRT and/or BT. Depending on the tumor stage, disease control and long-term survival are achievable (37–40). In a cohort of 1,322 patients with endometrial cancer treated with radiotherapy alone (EBRT and/or BT) for various reasons, the disease-specific survival at 5 years was 78.5%. Reported severe late morbidity (≥ grade 3) was as low as 3.7% for the combined treatment approach (40). In a smaller retrospective study, 74 patients with stage I and II endometrial cancer have been investigated. The majority of patients received a combination of pelvic EBRT and BT with curative intent, resulting in a 3-year progression-free survival of 68% with a median interval of 43.5 months (38). BT alone has been applied with curative intent, with excellent LC up to 100% in well-selected patients (41, 42).

For patients not able to undergo surgery and/or BT, the functionality of MR Linacs might have potential for improving EBRT. The suggested benefit of an oMRgRT and replanning over standard EBRT is the opportunity to truly adapt the treatment plan to the anatomy of the day. Variations in uterine position based on bladder or rectal filling can be visualized and accommodated rather than having multiple plans created ahead of time from which to choose the most appropriate plan of the day. As described earlier, for patients with LACC, the MR Linac treatment fields are limited in a cranial-caudal direction. The current available field lengths (Unity/Elekta: 22 cm; MRIdian/ViewRay: 24.1 cm) can be too limited for pelvic fields in tall patients, or if PAO elective radiotherapy is indicated. However, MR Linacs provide the possibility for boosting the uterus and any metastatic nodes in addition to elective EBRT when a BT boost to the uterus is not feasible. Daily MRI guidance and replanning allow for better targeting of the dose to the uterine cavity and extensions of the disease into the uterine wall and/or cervix while adapting for the variable positions of the sigmoid, small bowel, and bladder. Though the achievable target doses are not expected to be as high as with BT, a meaningful boost may be achieved dependent on volume and extensions of disease remaining after external beam. To date, there are no clinical cases/studies published reporting the early experience with this new treatment option, and therefore the potential gains remain theoretical.

### Vaginal Vault Recurrences

Vaginal recurrences can occur after treatment of cervical, endometrial, and vaginal cancers. Depending on the initial treatment of the primary tumor, treatment for recurrent disease may consist of surgery, chemotherapy, and/or RT (43, 44). When surgery is not an option, EBRT, BT, or both may be needed. The dose and fractionation will depend on the prior treatment. SBRT and especially IGBT show encouraging results (45). BT offers the most definitive boost treatment, and high LC rates can be achieved (45, 46). In an overview of 28 patients described by Fokdal et al., the 2 years LC rate was 92% (46). However, not all recurrences are amenable to BT salvage. An example may be a rectovaginal septum recurrence in close proximity to the rectal wall. In these complex situations, interstitial implants might be needed but are often not achievable, and the risk of fistula formation after treatment is high. In these situations, an external beam boost using the advantage of oMRgRT adjusting the plan to the daily anatomy with relatively homogeneous dose distributions may provide a good alternative. Utilizing isotoxic treatment planning for each fraction, tailoring dose away from the uninvolved rectum and other surrounding organs, and the avoidance of extremely high doses around the individual interstitial brachytherapy needles might result in less normal organ damage (including necrosis). Case example 2 demonstrates the first clinical experience with such a situation.

### Pelvic, Abdomen, Abdominal Wall Recurrences

Single or oligo recurrences of gynecological cancers at other locations in the abdomen (pelvic wall, abdominal wall, lymph node recurrences, and other soft tissue lesions) can be treated with surgery and/or radiotherapy to achieve long-term LC (47). Salvage surgery is not always possible, either due to unfavorable locations and/or anatomically challenging situations in case of repeated surgical interventions or patients unfit for surgery (48). Salvage irradiation can be used as an alternative to treat these recurrences (49). SBRT has curative potential in patients with recurrent gynecological malignancies (50). In a cohort of 30 patients treated with SBRT for metastases in the pelvis and/or the PAO region, 9 of 35 lesions treated with SBRT failed locally (26%), resulting in LC rates of 80 and 73% at 1 and 2 years and a 5-year survival of 42%. These results are promising in the setting of metastatic disease but also show that improving LC might have additional potential. In these situations, MRI guidance and online replanning might offer dosimetric gain, especially when SBRT, with the typical sharp dose gradients, is planned but highly mobile sensible OARs are in close proximity and vulnerable to injury. For first clinical experience, see *Case Example 2*.

## Treatment of Oligometastasis/Metastasis of Any Gynecologic Sites

### oMRgRT for the Management of Oligometastatic Disease

The concept of oligometastatic disease was first introduced in 1995 by Hellman and Weiselbaum (51), with the description of an intermediate state of metastasized disease between a locally confined and a widespread metastatic disease. The oligometastatic state was recently defined by an ESTRO-ASTRO consensus as one to five metastatic lesions where all metastatic sites must be safely accessible for curative intent treatment, with a controlled primary tumor being optional (52, 53). Early clinical studies showed an improvement in progression-free survival or overall survival (54–56) by the addition of metastases-directed therapy to standard-of-care systemic therapy in solid tumors. Today, this approach is supported by a large number of high-quality studies (57–59) and has rapidly gained attention in the field of radiation oncology as the proportion of patients receiving metastasis-directed therapy is constantly growing (60).

Several recent technology developments have facilitated the applicability of this concept: first, improved diagnostic imaging (e.g., PET-CT) enables an early detection of low disease burden. With the clinical implementation of high-precision local-ablative treatments such as SBRT, high LC rates with usually low toxicity can be achieved, while in parallel more effective systemic treatments have led to a prolonged overall survival of metastatic patients. Finally, we have improved the biological and clinical understanding of tumor biology; today genetic, molecular, or cellular analyses can help to tailor cancer treatments in the setting of precision medicine (61, 62).

SBRT is a local treatment modality that can be applied in few treatment sessions, allows simultaneous treatment of multiple targets at distant sites, and can be integrated into multimodality treatment regimen with minimal interference with systemic treatment delivery. However, current image-guided RT methods using on-board CBCT are limited due to the reduced soft-tissue contrast. It remains difficult to distinguish tumor from normal tissues, with the consequence that dose escalation strategies are not feasible in all anatomic regions, or generous target volume margins are applied to compensate for uncertainties in dose delivery and target coverage (61, 63). In this context, the application of oMRgRT marks the beginning of a new era. It allows direct visualization of the tumor and healthy tissues and provides real-time imaging during dose delivery. In addition, online ART allow to optimize dose escalation, while reducing dose to surrounding OAR on a daily basis. This technology offers the potential to further push the limits of local ablative treatments in the setting of oligometastatic disease.

### oMRgRT in the Management of Oligometastatic Lymph Node Metastases From Gynecologic Malignancies

Isolated lymph node metastases from gynecologic malignancies are considered a good indication for SBRT, as they usually occur within the pelvis or the PAO lymph node region (64, 65). SBRT can be applied in the setting of limited oligometastatic disease, with the aim of postponing or enhancing systemic therapies, or as an alternative to surgical resection (66). Patients are usually asymptomatic, as the disease burden is extremely low. In this setting, SBRT offers excellent tumor control rates with a low toxicity profile due to the small target volumes (67–69).

Obviously, this approach carries the risk of out-of-field local progression in other regional lymph nodes, which happens in 10–30% of patients (67). Locoregional progression could be prevented by using larger EBRT fields, which on the other hand might be limited due to overlapping volumes or treatment fields with previous radiotherapy areas and lead to higher morbidity rates (67). The fact that the risk of locoregional failure is low supports the rationale for the use of more limited field in patients with oligometastatic disease. A permanent remission can also be achieved by the iterative application of local interventions (70). Further studies are needed to identify specific biomarkers for accurate patient selection of true oligometastatic disease and determine the optimal way to integrate and sequence SBRT in multimodal treatment approach (53).

The effectiveness of SBRT on LC is clearly associated with a dose-response correlation; higher biologically equivalent dose (BED) leads to better tumor control (71, 72). For lymph node metastases, 5-year LC rates of uterine, cervical, and ovarian cancer range from 70 to 97%, and favorable disease-free survival and overall survival are reported in retrospective series (64, 68, 70, 72–77). Lymph node metastases of gynecologic malignancies are often located in the pelvis or abdomen, where conventional SBRT using CBCT image guidance yields relatively poor soft tissue contrast. Hence, it may be difficult to deliver a sufficient dose to the tumor because it is challenging to identify the interface between the lymph node metastasis and surrounding healthy tissues (e.g., bowel), even if a steep dose gradient can be achieved with SBRT. In these clinical scenarios, oMRgRT offers significant advantages, as it allows a direct visualization of the metastases on MRI and enables margin reduction or dose escalation strategies by using online ART and automated gating systems (78). Comprehensive documentation of treatment outcomes of the first successfully delivered treatments will confirm whether or not it will translate into a clinical benefit. There is no data from large series available yet.

Early experiences with MRg SBRT of lymph node oligometastases of other primary tumors show promising results (61, 65). A dosimetric comparison of the dose coverage and compliance to dose constraints of an MR Linac workflow with a CBCT workflow in lymph node SBRT showed a lower number of unplanned violations of high-dose criteria using the adaptive MRg treatment planning at comparable target dose coverage (79). oMRgRT can provide correction for inter-fraction setup uncertainties, changes in size and shape of the tumor, as well as the anatomical alignment to OAR. To fulfill this task, several plan adaptation strategies are available on MR Linacs (80), which vary from simple weight optimization or multileaf collimator shifts to advanced full online adaptive replanning where a completely new treatment plan is generated. The goal of daily plan adaptation can be to improve target coverage, OAR sparing, or both (78). A recent study of Winkel et al. (80) showed that in patients with oligometastatic lymph node metastases, the most advanced optimization method, using a full online replanning, performs as good as pre-treatment planning, yields the most favorable dosimetric values, and can be performed within a reasonable timeframe.

### oMRgRT for the Management of Oligometastatic Distant Metastases From Gynecologic Malignancies

A small subgroup of patients diagnosed with oligometastatic distant disease may benefit from local metastases-directed therapy, even if treatment options have traditionally been limited to systemic therapy with palliative intent in this clinical setting (68). Lazzari reported on the outcome of SBRT in oligometastatic ovarian cancer (74). SBRT in oligorecurrent or oligoprogressive disease in intensively pretreated patients (median of three prior systemic therapy regimens) showed excellent LC rates without any grade 3 or 4 acute or late toxicity. The median systemic treatment-free interval after SBRT was 7.4 months, and more than one-third of patients were still disease-free at 1 year after SBRT. In this context, SBRT was able to postpone systemic therapy and allowed “drug holidays” in a heavily pretreated group of patients. Since the failure pattern was predominantly out of field (75%), multiple SBRT courses were used as a salvage option in case of subsequent recurrence.

In the treatment of distant metastases (liver, lung, bone, soft tissue), higher BED correlates with better LC rates (81, 82). Kunos et al. (81) achieved an LC rate of 100% in metastatic gynecologic cancers with a prescription dose of 24 Gy in three fractions (70% isodose), and Mesko et al. (82) reported an LC rate of 83% after applying a median dose of 40 Gy in five fractions. In contrast, Lazzari et al. (74) reported an LC rate of only 70% for distant metastases after SBRT with 24 Gy in three fractions, while lymph node metastases reached higher LC rates of 81% with the same fractionation. A large retrospective multicenter analysis of SBRT of 449 ovarian cancer lesions (76) found that an age of ≤60 years, a PTV size of ≤18 cm3, lymph node disease, and a BED (α/β10) of >70 Gy were independent predictive factors of complete response on multivariate analysis. SBRT is technically feasible in all anatomic regions. The fractionation and prescription dose vary widely based on tumor-related parameters (lesion size, proximity to vulnerable OAR, organ and tumor motion) and if the target lies in a previously irradiated field. Breathing motion and changes in the filling status of surrounding OAR can present a challenge (83). oMRgRT can improve the feasibility of delivering SBRT for oligometastatic distant disease, enabling dose escalation. In addition to the advantages of online ART, MR Linacs allow for a direct visualization of the target during treatment delivery. The 0.35T MR Linacs can automatically gate the beam by using real-time anatomy structure tracking at a rate of eight images per second (84). This eliminates the need for invasive implantation of fiducial markers and the addition of an ITV to account for intra-fractional motion, leading to reduced healthy surrounding tissue irradiation (85, 86).

Appropriate patient selection is key for success. The treatment of oligometastatic distant disease with a limited number of lesions can represent a spectrum of clinical scenarios, which are associated with different prognoses and might require different treatment strategies. In a recently published ESTRO-EORTC consensus, an attempt was made to characterize and classify the different possible stages of oligometastatic disease (53). The classification differentiates between a true oligometastatic disease and an induced oligometastatic condition, where patients had a prior history of polymetastatic disease. Furthermore, oligorecurrence, oligoprogression, and oligopersistence were classified, considering whether the oligometastatic disease was diagnosed during a treatment-free interval or under active systemic therapy. However, to date no biomarkers are available to identify patients with true oligometastatic disease, and the sole presence of limited disease is sometimes difficult to interpret (53). In a retrospective analysis, patients with limited disease burden of ovarian cancer (stage I-II, no residual tumor after first surgery, fewer previous systemic therapies, ≤2 lesions treated, time since last chemotherapy ≥7 months) had a better outcome than patients undergoing SBRT after failure of multiple lines of chemotherapy or in case of induced oligometastatic disease (74). These patients may not have been in a truly oligometastatic state at the time of SBRT. Therefore, further studies are needed to establish adequate selection criteria and to define the role of SBRT in the multidisciplinary treatment strategy of oligometastatic distant metastases and its influence on survival outcome.

### oMRgRT for the Management of Oligometastatic Paraaortic Relapse

A minority of patients develop oligometastatic relapse in the PAO region after curative surgery or pelvic (chemo)radiation for primary gynecological cancers (especially cervix or endometrial origin). For these patients, PAO irradiation with or without systemic treatment can be offered as salvage option (87). PAO irradiation can be applied as regional elective treatment including simultaneously integrated boosts (Sib) to macroscopic nodal metastases or as localized approach (especially SBRT) for macroscopic disease alone (88). Dose levels needed to achieve control are 45–50 Gy in 25–28 fractions for elective volume and a dose range of 50–65 Gy for macroscopic disease (88). The proximity of these nodes to the bowel is a dose-limiting factor. Severe duodenal morbidity was reported after PAO irradiation using Sib to nodes in the upper abdomen (87, 89). MR Linacs might be the technology of choice in these situations as daily visualization of the anatomy together with the possibility for online ART allows for a broader therapeutic window with better tailoring of the dose to the metastatic nodes and away from the surrounding bowel (duodenum) loops (see *Case Example 3*).

## Clinical Cases

### Case Example 1: Primary Cervix Cancer; MR Linac Boost to Primary Tumor (**Figure 3**)

A 54-year-old patient with FIGO stage IVA cervical cancer

**Figure 3:**
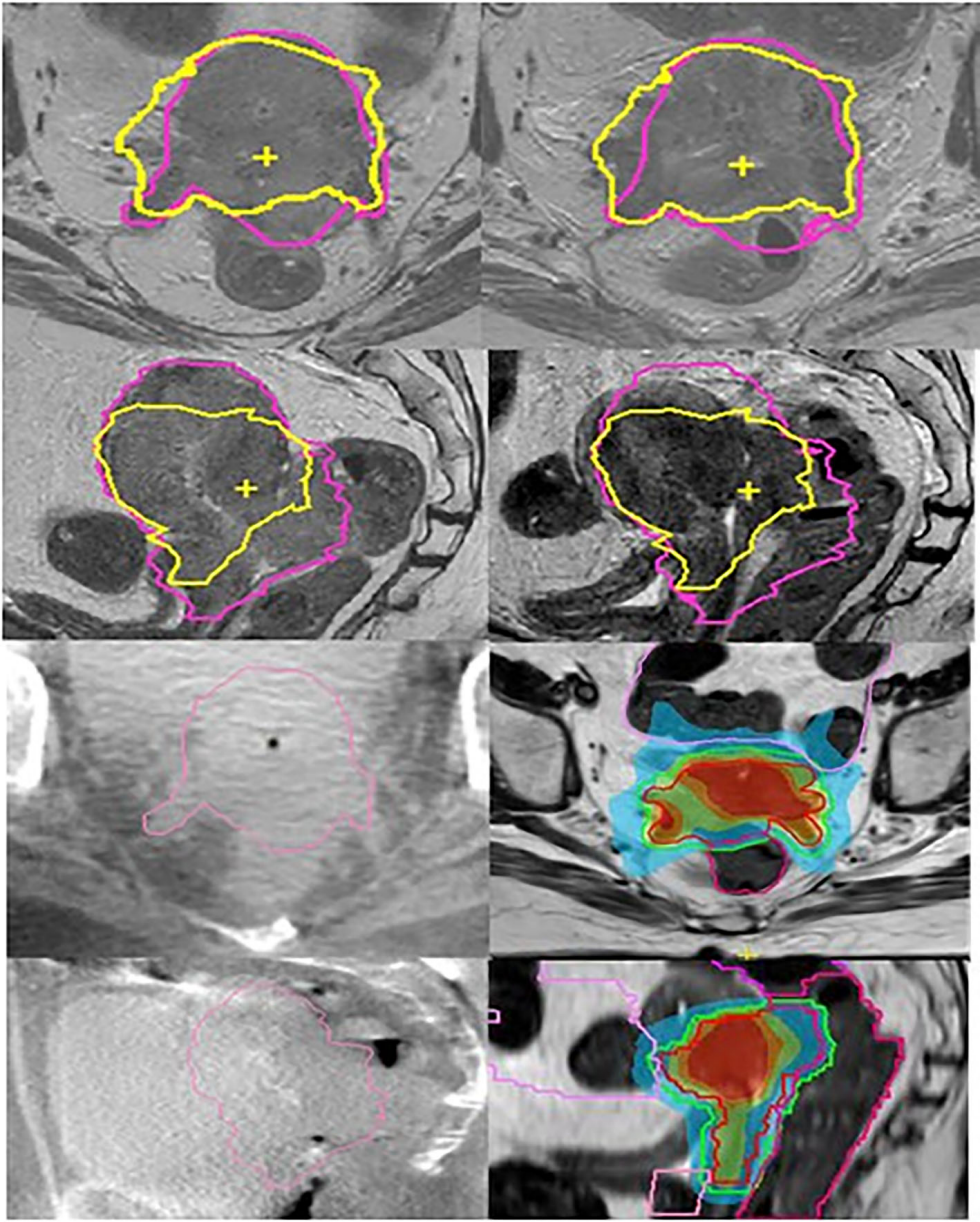
Cervix cancer; MR linac boost of HR-CTV after pelvic EBRT. Left column (top to bottom) transversal and sagittal T2 weighted MRI at time of treatment planning and on-board CBCT scans in the first week of elective EBRT. Right column (top to bottom) MRI scans after 32.4 Gy of elective EBRT and first. MR Linac boost plans. For comparison reasons, the initial and boost HR-CTV contours are shown on the MRI scans (pink at time of treatment planning and yellow after 32.4 Gy EBRT). On the CBCT scans initial HR-CTV is shown. For the MR Linac boost plan, the online delineation of the first fraction for HR-CTV and rectum is shown. The images show the improved visualization of MRI compared to CBCT.

Primary tumor infiltrated the distal parametrial tissue, rectovaginal septum, upper vagina, rectal wall, and bladder mucosa and was associated with bilateral hydronephrosis. Patient’s history included brainstem infarction with persistent hemiplegia and need for anticoagulation. Multidisciplinary recommendation was to offer a curative treatment. EBRT was delivered with VMAT (45 Gy in 25 fractions to tumor and lymphatic drainage) with bilateral Sib (2.35 Gy per fraction) for two positive obturator nodes. Concurrent chemotherapy could not be administered due to severely impaired kidney function and comorbidity. Initial plan included a BT boost (four HDR fractions, aiming at a total D90 HR-CTV of 90 Gy EQD2α/β=10).

After 32.4 Gy of EBRT, repeated MRI showed only minor tumor regression and persistent tumor invasion in the rectum and bladder. Tumor volume was reduced from 174 to 118 mm3, and largest dimension was still significant (from 93 to 80 mm). Therefore, BT was no longer considered feasible, and a boost was delivered using oMRgRT instead.

The boost was delivered in four IMRT fractions using an 11-field beam arrangement. Planning was done using an isotoxic approach with priority given to OAR dose constraints over target coverage. The HR-CTV and relevant OAR were re-contoured before each fraction using the daily MRI and online ART was performed.

The pelvic EBRT dose (45 Gy or 44.25 Gy, EQD2α/β=10) was added to the dose from the four online adaptive plans to calculate the cumulative dose, which was as follows: D90 HR-CTV: 76.4Gy EQD2α/β=10 (i.e., 6.0 Gy, or 8.1 Gy, EQD2α/β=10 per fraction), and OAR doses were bladder D2cc: 90.9, rectum D2cc: 70.0, sigmoid D2cc: 47.3, and bowel D2cc: 74.9 Gy EQD2α/β=3. Although D90 HR-CTV was below the recommended dose (D90 ≥ 90 Gy EQD2α/β=10), using this stereotactic planning approach at least part of the HR-CTV received this dose with V90GyEQD2α/β=10 = 19%, and V85GyEQD2α/β=10 = 64%. Our institution approach (UMCU) for CBCT Linacs would have allowed for a total D90 HR-CTV of 70 Gy EQD2α/β=10 using VMAT with uniform target dose distribution.

Treatment was well tolerated without unexpected early toxicity. First follow-up including MRI-based response evaluation will be performed 3 months after treatment.

### Case Example 2

A 68-years-old patient diagnosed with FIGO stage IIIB grade 2 endometrial cancer, treated with laparoscopic hysterectomy and bilateral salpingo-oophorectomy. On the treatment planning CT scan and MRI, recurrent tumor was detected in the vaginal vault, and additionally a second lesion was seen in the anterior abdominal wall (most likely a laparoscopic port site recurrence). Biopsy of both lesions confirmed metastases from endometrial cancer. Patient was considered ineligible for additional surgery due to comorbidities including severe obesity. Definitive radiotherapy was planned, consisting of sequentially oMRgRT SBRT for the abdominal wall metastasis (35 Gy in five fractions), followed by pelvic EBRT with VMAT on a conventional Linac (45 Gy in 25 fractions) and finally a sequential oMRgRT boost to the vaginal vault recurrence (28 Gy in four fractions). Vaginal vault BT was considered but not deemed feasible since a complex interstitial approach under anesthesia would have been necessary and was not doable due to comorbidity.

**Figure 4:**
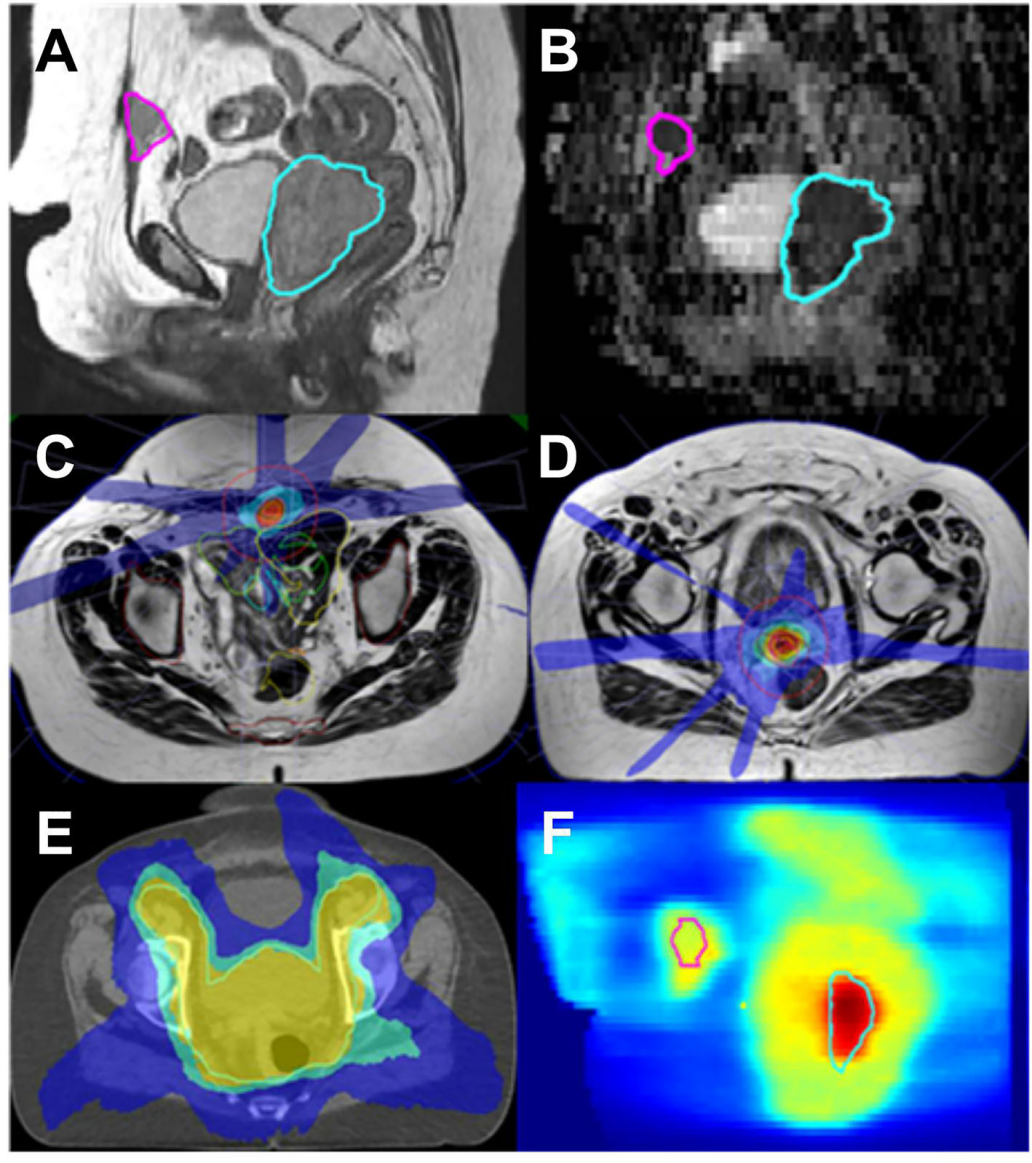
Endometrial cancer; MR Linac SBRT for concurrent vaginal vault recurrence and anterior abdominal wall metastasis. **(A)**: T2 sagittal **(B)**: ADC map derived from diffusion weighted acquired with MR Linac. Abdominal metastasis in pink and vaginal vault recurrence in blue. Target and OAR are clearly visualized on the MR images allowing for daily adaptation. **(C, D)**: Typical daily MR Linac plans for both lesions (isodoses red 110%, orange 100%, blue 25%). **(E)**: elective EBRT plan on planning CT (yellow 95%, green 82%, blue 52%) **(F)**: overlay of elective and boost plans (range 0–70 Gy physical dose).

A composite was done for each of the three radiotherapy courses (see **Figure 4**).

An isotoxic approach was used, giving the priority to OAR dose constraints during the planning process. The initial prescription (sum of EBRT and four boost fractions) to the vault recurrence (HR-CTV) was 91.9 Gy (D90%, EQD2 α/β 10), whereas the total dose delivered to this volume based on the sum of daily online planning was 82 Gy (D90%, EQD2 α/β 10). For D2 cc bladder and rectum, the pretreatment and online doses were 84.9 Gy/73.5 Gy and 68.3 Gy/73.7 Gy (D2cc, EQD2, α/β 3), respectively. The differences in pretreatment and online dose were mainly caused by variations in rectum positions and filling status, which in our isotoxic planning approach resulted in a reduced target dose.

For the abdominal wall metastasis, the SBRT planning aim was 35 Gy in five fractions to 95% of the target. For both pretreatment and online plan, the GTV35Gy (EQD2 α/β 10 = 50Gy) had a median value of 100%.

MRI done 3 months after treatment showed no residual tumor in both locations and no evidence of disease progression. So far, patient did not report unexpected or grade ≥3 treatment toxicity.

### Case Example 3: Ovarian Cancer; Paraaortic Oligometastatic Relapse

**Figure 5:**
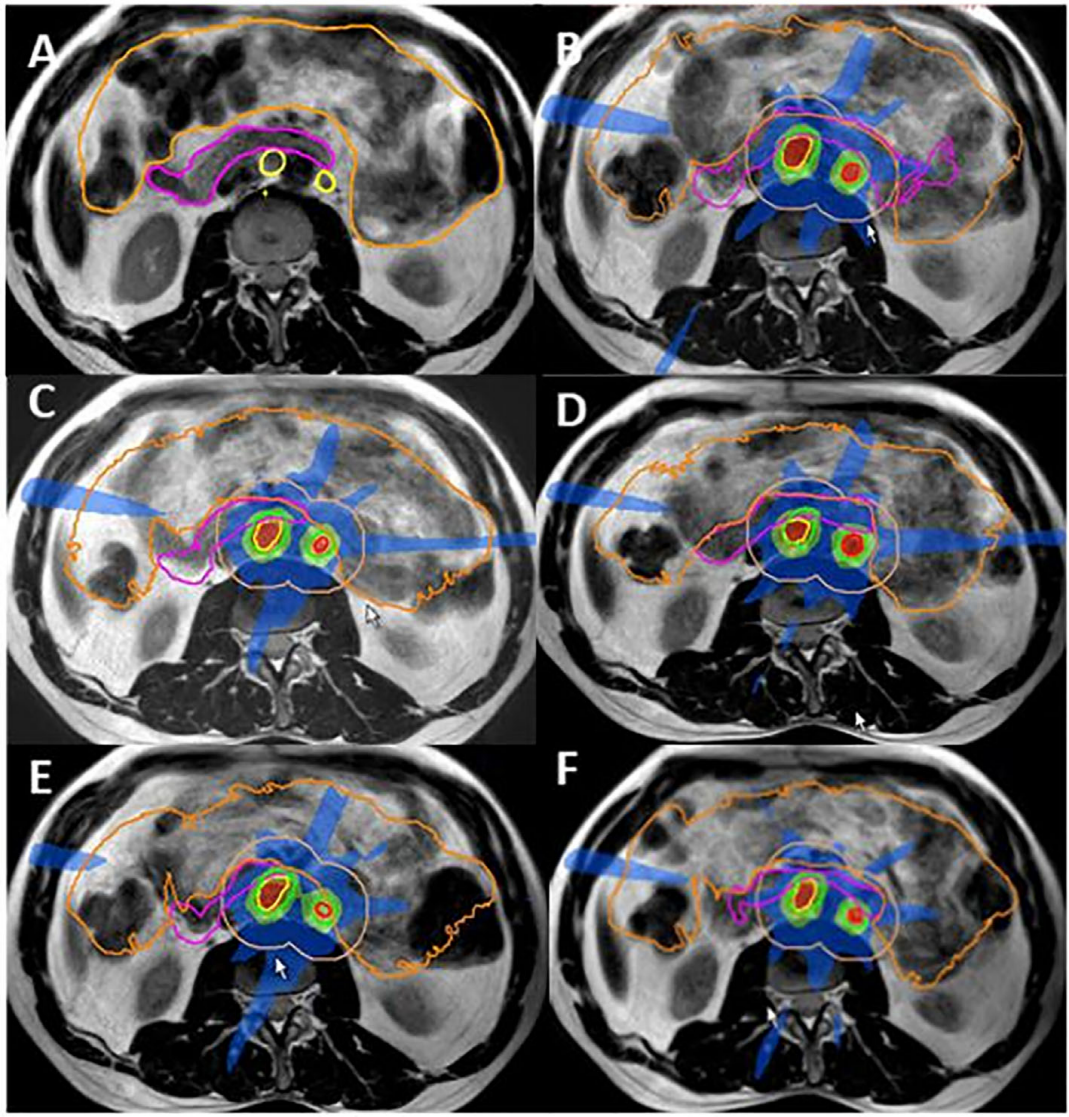
Ovarian cancer MR Linac SBRT for PAO oligometastatic relapse. **(A)** Contours delineated for pretreatment planning GTV1 and GTV2 yellow; duodenum pink, bowel orange. **(B–E)**: Online dose distribution for fractions 1–5, GTV1 and GTV2 (red) and PTV1 and PTV2 (green) with 2 cm ring for online contouring and planning guidance orange. Within the ring structure, target structures were manually adapted for the duodenum (pink) and bowel bag (orange) after an initial automated deformation of the contours; **(F)** individual dose distribution with dose levels shown as percentage of 35 Gy (5 × 7 Gy) prescribed dose red 110%, orange 100%, light green 75%, and blue 50%.

A 51-year-old patient presenting with PAO relapse from ovarian cancer after previous treatment including primary surgery, chemotherapy, and targeted treatments at the time of 2nd and 3rd relapse. Surgery or further systemic treatment was not considered feasible at this time. Three PAO nodes in very close proximity to the duodenum were treated with oMRgRT. Five fractions were delivered using daily online ART to create nine-field IMRT plans with a stereotactic dose distribution. GTV-PTV margin was 3 mm. Dose prescription was: GTV V35Gy = 100%, PTV V35 Gy > 95%, PTV D0.1cc < 47.25 Gy. Constraints for the duodenum were D0.5cc <35Gy and D5cc <25Gy (**Figure 5**).

Total target and OAR dose as calculated for the pretreatment plan and the five online adaptive plans for target lesions and the duodenum are shown in **Table 1**. The dose distribution had to be balanced between adequate target dose coverage and OAR constraints, resulting in a slightly lower dose for the three GTVs and a slightly increased dose for the duodenum. **Figure 5** shows target delineation and dose distributions of the five fractions.

**Table 1:** Case example 3: DVH parameters for planned *versus* accumulated dose from online adaptive treatment plans (total dose delivered with five fractions SBRT).

	Dose Prescribed (Gy)	Dose Delivered (Gy)	EQD2* Per prescription (Gy)	EQD2* Delivered (Gy)
**GTV1 D100%**	34.0	33.2	47.5	46.2
**GTV2 D100%**	34.7	34.3	49.0	48.2
**GTV3 D100%**	37.6	34.8	54.8	49.2
**Duodenum D0.5cc**	34.8	34.9	69.2	69.7
**Duodenum D5cc**	28.8	29.7	50.5	53.2

*For EQD2 calculations a/ß = 10 for GTVs and a/ß = 3 for OAR was applied.

At 2 months post treatment, patient is in good condition without any early treatment toxicity.

## Discussion

The introduction of oMRgRT in radiation therapy clinics brings opportunities to improve the accuracy of EBRT for the treatment of mobile soft tissue primary tumors and distant metastases. Combining high-quality on-board imaging and adaptive therapy capabilities is of high value for soft tissue tumor prone to have significant inter-fraction or intra-fraction motion.

Gynecologic tumors fit in this category of cancers as the surrounding OAR can cause considerable target deformation and position changes. Surrounding pelvic organs can move closer to the target compared to the original reference plan, and finally tumor volumes and shapes often change significantly during treatment.

During the last two decades, repeating MRI studies through the course of treatment and implementing an adaptive treatment planning strategy have led to improved BT treatment outcomes (5). IGABT has been shown to be associated with improved tumor control and better survival (7, 9, 10, 31). The early experience with the use of MR Linac systems, described in this manuscript, demonstrates that oMRgRT has the potential of improving EBRT outcomes as well (15, 26, 61, 65).

LACC is a key example of tumors which could benefit from the use of oMRgRT, given the better soft tissue visualization provided by on-board MRI compared to CBCT, and the possibility to correct for any target motion and changes in surrounding organs position on a daily basis. Single-institution experience as described in this manuscript indicates that it is possible to use MR Linacs for pelvic radiation during a course of curative treatment for cervical cancer. The restricted length of treatment fields of the current MR Linac systems, however, brings limitations when extended field RT is required. In the future, solutions like VMAT combined with a tomotherapy approach would be of utmost interest to allow for treatment of larger volume. The treatment field size limitation of MR Linacs currently prohibits the use of MRg when either the high common iliac or the PAO nodes need to be treated, which is frequent in the management of LACC. This is a clinical situation where better conformity of radiation dose distribution is especially needed, since the radiation tolerance of the surrounding organs (small bowel loops, particularly the duodenum, kidneys) is low (88), while high dose of radiation is needed for tumor control. Better dose conformity is especially needed when chemotherapy is combined with extended field RT or in cases of oligometastic PAO disease. In both situations, patients will benefit from more conformal dose distributions that allow for dose escalation with a broader therapeutic window. When multiple targets need to be treated (synchronous treatment of primary tumor and multiple affected nodes or oligo metastases), MR Linac treatments, with the possibility to perform adaptive plan daily and use smaller treatment margins, are of interest (65).

While MR Linac systems (Unity/Elekta Sweden or MRIdian/ViewRay Cleveland, OH, USA) were the first radiation delivery systems with online adaptive capabilities, there is now a CBCT-based Linac (ETHOS, Varian, Paolo Alto, CA, USA). The strength and limitations of each technology will become clearer as we gain more clinical experience using these systems. Inferior soft tissue contrast might be a limiting factor to perform ART with the CBCT Linac option.

Online ART requires recontouring and replanning while the patient is on the table; therefore, extended treatment time is a concern for the clinical implementation. More experience will be needed to prove if ART could lead to improved treatment outcomes and if the benefit of this treatment approach outweighs the cost on department resources. Successful attempts to automate delineation and increase speed of planning software would affect this balance.

When treating gynecological cancers, radiation boosts are frequently used to treat the primary tumors or central local recurrences. BT (preferably IGABT) is the treatment modality of choice to deliver these boosts and should be applied whenever feasible. However, there are some frail patients for whom invasive procedures cannot be done, and there are clinical situations where the extension of the disease is unfavorable for an adequate implant. In these situations, oMRgRT can be used to deliver highly conformal external beam boosts. As a starting point, traditional dose constraints that are used for hypofractionated BT SBRT should guide the selection of oMRgRT boost dose and fractionation prescription (26).

Based on treatment planning comparisons and initial clinical experience, the therapeutic window of oMRgRT boosts is not as good as optimal BT but compares favorably to non-adaptive CBCT-guided plan.

In conclusion, oMRgRT provides options for the delivery of more conformal therapy using an ART approach for patients with gynecological cancers in different disease stages. Future clinical experience will confirm if the expected gain in treatment conformity will translate into improved clinical outcomes. For the management of central pelvic disease, BT is the most conformal treatment technique to deliver an ablative dose to the tumor, and oMRgRT boosts should not replace BT in situations where it is feasible, due to the well-documented success rates achieved with BT (10).

## Author Contributions

All authors listed have made a substantial, direct, and intellectual contribution to the work, and approved it for publication.

## Conflict of Interest

The authors declare that the research was conducted in the absence of any commercial or financial relationships that could be construed as a potential conflict of interest.

## Publisher’s Note

All claims expressed in this article are solely those of the authors and do not necessarily represent those of their affiliated organizations, or those of the publisher, the editors and the reviewers. Any product that may be evaluated in this article, or claim that may be made by its manufacturer, is not guaranteed or endorsed by the publisher.
